# The Great Battle

**Published:** 1862-11

**Authors:** 


### THE GREAT BATTLE

Between a true liberty and despotism; between good and evil; between light and darkness; between truth and error; between holiness and sin, is in progress: not as yet at its height, perhaps, for there are omens of troublous times ahead, and the weapons of that warfare should be well understood by every soldier whose ambition is to earn a name and a crown. The weapons are two: truth and love. They are to be burnished, brightened, and sharpened, by the proper cultivation of the head and heart; by the exercise of thought and feeling: thought to cleave, feeling to melt. Thought forges ideas; feeling goes out in acts. Thought is power; feeling is power. Both throw out great truths; and those who can best express those great truths in words—words which shall strike home those truths upon the heart and intellect, leaving an impress there, deep, clear, distinct, abiding—these are the noble men who are forging weapons for that war, to be used, if not by themselves, yet will be by some equally noble brother who comes after, to fill the vacated place in the ranks.

